# Plant-based macronutrients and micronutrients in combating osteoporosis: research insights and molecular pathways

**DOI:** 10.1039/d6ra02084d

**Published:** 2026-07-18

**Authors:** Madhusri Roy, Gouranga Dutta, Amrita Das, Smriti Singha Roy, Biplab Debnath, Mayukh Jana, Abimanyu Sugumaran

**Affiliations:** a Department of Pharmaceutical Technology, Bharat Technology Uluberia Howrah 711316 India mayukhjana@gmail.com; b Department of Pharmaceutics, Eminent College of Pharmaceutical Technology Barasat 700126 India; c Department of Pharmaceutical Sciences, Sushruta School of Medical and Paramedical Sciences, Assam University Silchar Assam India abipharmastar@gmail.com abimanyu.s@aus.ac.in

## Abstract

Osteoporosis is a chronic metabolic bone disease that leads to decreased mineral density and causes damage to bone microarchitecture. Elderly persons are more susceptible to bone fractures and have reduced bone strength due to osteoporosis. Conventional pharmacological treatments are the key therapies, but increasing evidence highlights the potential of nutritional interventions to support bone health. Macronutrients, especially proteins from soy, legumes, nuts, seeds, and whole grains, supply essential amino acids vital for type I collagen synthesis, osteoblast differentiation, and bone-forming signalling pathways, promoting bone regeneration and structural integrity. Micronutrients from green leafy vegetables, including magnesium, potassium, vitamin K, vitamin C, zinc, and boron, contribute to mineral homeostasis, enhance calcium absorption, manage oxidative stress, and impact key pathways in bone remodeling, such as RANKL/OPG, Wnt/β-catenin, estrogen modulation, and antioxidant mechanisms. This review examines the current research on the usefulness of macronutrients and micronutrients from numerous plant sources in promoting bone tissue regeneration and reducing dependence on synthetic anti-osteoporotic treatment. It highlights their roles as sustainable, low-risk supplements to traditional osteoporosis treatments by integrating molecular insights with dietary knowledge.

## Abstract 2

Osteoporosis is a chronic metabolic disease causing low bone mineral density and microarchitectural damage. However, plant-based macro and micronutrients offer a promising preventative strategy against this deterioration.

## Graphical abstract

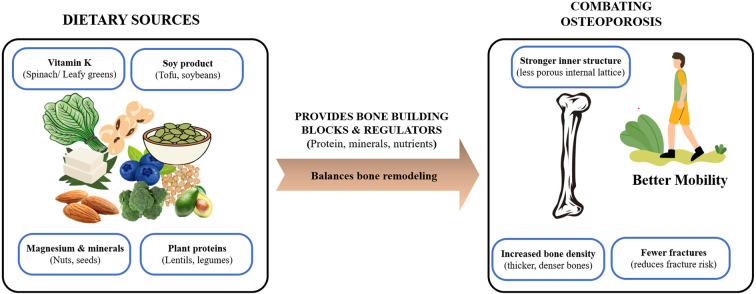

## 1. Introduction

Osteoporosis is generally defined as a skeletal disorder where the bones actually lose their density, resulting in the bones becoming weaker and more fragile, which may cause them to break or fracture in case of any minor accident or sudden fall.^1^ Osteoporosis is the most common metabolic bone illness and is becoming an increasingly serious issue, impacting 200 million people globally. Osteoporosis is far more common in women, particularly after menopause, due to the rapid decline in estrogen, which promotes bone loss. Globally, approximately one in every three women over the age of 50 will sustain an osteoporosis-related fracture, compared to one in every five men. In the United States, 19.6% of women over the age of 50 have osteoporosis, compared to 4.4% of males.^2^ Globally, women account for over 80% of all osteoporosis cases.^3^ Some nations, such as Finland and Slovenia, have the highest reported osteoporosis rates, with a large gender discrepancy in prevalence. Even in countries with reduced frequency of osteoporosis, overall numbers remain high due to population size (*e.g.*, China and India).^4^

Osteoporosis is frequently undertreated and underdiagnosed, partly because it is a clinically quiet condition until it shows as a fracture. It is critical to recognize the disease and treat it appropriately, both medically and nonmedically.^5^ The bone mineral density decreases consistently with age, so there is a high chance of having more bone fractures. According to some studies, it has been found that women have three times higher risk of having fractures than men because of their low bone mass.^3^ In the case of women, when there is a lack of estrogen (mainly during menopause) in the body, it causes bone turnover, which increases RANKL and lowers osteoprotegerin (OPG), resulting in the occurrence of osteoclast.^6^ From various data, it has been found that one in every three women and one in every five males over the age of 50 will develop an osteoporotic fracture during their lifetime.^7^ Even though there have been improvements in drug treatments, the long-term treatment of osteoporosis needs a wider approach that helps bones regenerate, lowers the risk of fractures, and makes the whole skeleton stronger. In the last ten years, increasing evidence has shown that diet is really important for bone health, not only as a way to help but also to treat it. Micronutrients, like vitamins, trace elements, and macronutrients, like proteins, omega-3 fatty acids, and essential minerals, have been shown to change signaling pathways like Wnt/β-catenin, RANKL-OPG, and osteoblasts and osteoclasts activity. They can also make the bone matrix more mineralized. Bioactive chemicals that are found in nature have many benefits, such as being antioxidants, anti-inflammatory, hormone-modulating, and anabolic. These benefits may help keep bone turnover going and encourage regeneration.^8,9^ Numerous experimental and clinical investigations support the use of these nutrients as standalone treatments, adjunct therapies, or components of sophisticated biomaterial scaffolds for bone tissue engineering. Nutraceuticals and functional dietary components may enhance bone architecture and reduce disease development, according to these studies.

In regard to this growing information, nutrient-based mechanisms and their translational relevance must be understood. This review consolidates existing data on macro- and micronutrients pertinent to osteoporosis care, their natural sources, mechanisms of action, and their established roles in enhancing bone production and regeneration. This review seeks to elucidate the therapeutic potential of nutrition-driven bone health initiatives by combining molecular insights with clinical and experimental data while also identifying prospects.

## 2. Pathophysiology of osteoporosis

**Fig. 1:**
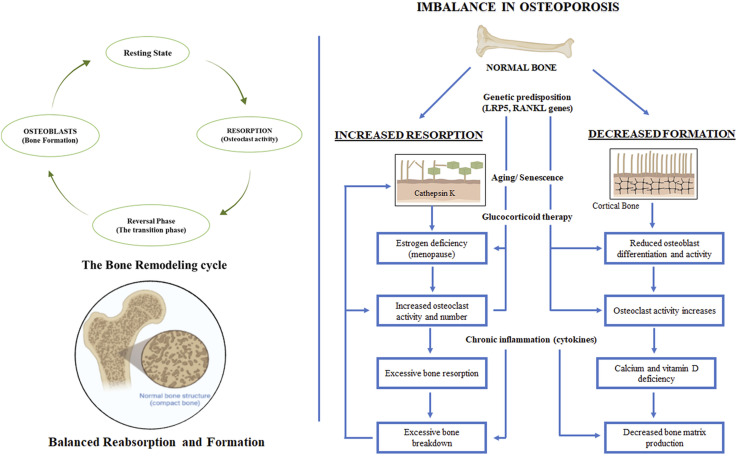
Pathophysiology of osteoporosis.

Osteoporosis is caused by an imbalance in the normal bone remodelling process, which requires an exact balance between osteoclast bone resorption and osteoblast bone creation to maintain bone mass and structural integrity. In healthy bone remodelling, osteoclasts resorb old or damaged bone, while osteoblasts lay down new bone matrices during the reversal and creation stages. This balance maintains steady bone mass and skeletal strength.^10^ The reasons include aging, hormonal changes (especially estrogen deficiency in postmenopausal women), chronic inflammation, genetic or epigenetic alterations, poor calcium and vitamin D intake, sedentary lifestyle, excessive alcohol use, and endocrine disorders, such as hyperparathyroidism.^11^ These lead to the deterioration of bone density and quality, making bones weak and prone to fractures, even with minor falls or accidents, particularly in the spine, hip, or wrist. Consequently, such injuries can result in reduced mobility and independence. Vertebral compression fractures cause height loss and spinal abnormalities, such as kyphosis.^11,12^ Moreover, osteocytes, the predominant bone cells, significantly regulate both osteoclasts and osteoblasts, and they may actively facilitate bone resorption under various situations.^13^ Bone remodelling consists of finely controlled cycles of bone resorption and creation mediated by osteoclasts and osteoblasts.^14^ Osteoclasts release enzymes, such as cathepsin K, and use protons to break down bone mineral, releasing calcium into the bloodstream. This process is started by the RANK/RANKL/osteoprotegerin signaling pathway, which controls osteoclast development and activity (Fig. 1).^9,15^ Osteoblasts, on the other hand, form the bone matrix and encourage mineralization using proteins like collagen and enzymes.^16–18^ Hormones, such as estrogen, play an important role in bone cell function by influencing gene expression through estrogen receptors, boosting osteoblast activity, and suppressing osteoclasts. Reduced estrogen levels during menopause upset this balance, causing great bone resorption and decreased creation, resulting in bone density and mass loss.^19^

The reduced estrogen levels following menopause contribute to increase osteoclast-mediated bone resorption, leading to lower bone mineral density and increased skeletal fragility in women. Estrogens help to monitor the osteoclast, which is the bone-breaking cell, by increasing the production of osteoprotegerin (OPG) and reducing the effect of osteoblast, also known as Receptor Activator of Nuclear Factor kappa-B Ligand (RANKL).^20^ The loss of estrogen during menopause causes various inflammations in the body. The estrogen deprivation in postmenopausal women produces a major increase in pro-inflammatory cytokines (such as IL-1, IL-6, and TNF-α), which accelerate inflammation and bone decay.^21^ Unlike women, men have a decline in bone density not because of chronic inflammation and hormonal changes. The underlying processes vary from those seen in women after menopause.^22^ Osteoporosis in males is often attributed to age-related declines in testosterone, chronic inflammation, oxidative stress, and comorbidities, such as hypogonadism, alcoholism, and prolonged glucocorticoid therapy.^23–25^ As men age, their testosterone and estrogen levels decline, leading to progressive bone loss and heightened inflammation, although at more gradual rates than in women.^23^

A major cause of osteoporosis is chronic inflammation and oxidative stress in both men and women, which disturbs the equilibrium of osteoblasts and osteoclasts, exacerbating bone fragility.^26^ As people age, oxidative stress increases, leading to the formation of reactive oxygen species (ROS).^27^ Oxidative stress arises when the body's antioxidant defence mechanisms and the production of reactive oxygen species (ROS) are out of balance during osteoporosis. Excess ROS promotes bone breakdown by increasing the activity of bone-resorbing cells (osteoclasts) and decreasing osteoblast production, resulting in bone loss.^26^ Due to ROS, the working of osteoblasts slows down, and the lifespan of the osteoclasts increases, which makes the bones much weaker over time.^27^

Another reason is the lack of vitamin D and calcium, which makes the bone condition much worse. Because our body keeps an excessive amount of calcium to maintain normal blood pressure, the release of parathyroid hormones (PTH) increases. This PTH sends a signal to the osteoclast to increase its working ability and to transfer the calcium from the bones to the blood.^28^ Some other reasons that worsen the bone health are not moving enough, *i.e.*, when we do not walk or move more or put any stress on our bones, our bones get weak very easily. Furthermore, when there is continuous inflammation in our body due to any disorder, it also has an impact on bone health. Some medications, such as steroids, when taken for a long period on a daily basis, worsen the condition of the bones and make it more prone to fractures.^29^ Long-term usage of steroids (glucocorticoids) can result in osteoporosis. For example, patients taking glucocorticoids to treat chronic inflammatory or autoimmune illnesses may develop glucocorticoid-induced osteoporosis. According to research, fractures can develop in 30–50% of persons taking continuous glucocorticoid medication.^29^ This occurs because glucocorticoids increase the activity of osteoclasts while decreasing the number and function of osteoblasts, resulting in relatively weak bones.^30^ Long-term glucocorticoid therapy, such as for asthma or rheumatoid arthritis, can result in glucocorticoid-induced osteoporosis, with up to 30–50% of chronic users developing osteoporosis.^31^ This causes trabecular thinning, where the honeycomb-like structure inside the bones, which has the ability to keep the bones strong, gets destroyed. This trabecular thinning occurs mainly in weak spots, such as the wrist and spine. It causes holes inside the bones, which make the bone more prone to fractures and also reduce the overall density and mass of the bone.^32^

## 3. Current treatment strategies and their limitations

Osteoporosis is usually treated using two types of medication, one that slows the breakdown of bone and the other that builds the bones faster. The medication used in the case of osteoporosis is bisphosphonates, which include risedronate and alendronate. The working mechanism of these drugs is to attach to the mineral part of the bone, which is known as hydroxyapatite, and send a signal to cause the apoptosis of the osteoclast, thereby preventing the breakdown of the bones.^33^ Raloxifene is one example of a selective estrogen receptor modulator (SERM) that mimics the effects of estrogen on bone health.^34^ Another way of treating osteoporosis after menopause is hormone replacement therapy (HRT), where the estrogen level is maintained to slow down the bone breakdown process and hasten the growth process of the new bones.^35^ Denosumab is another drug that is used for the treatment of osteoporosis. The mechanism of action of denosumab a bit different from other drugs. It works by blocking the signal of RANKL, which stops the process of the osteoclasts and improves bone health.^36^ The analogues of parathyroid hormones are also used for the treatment by stimulating the activity of osteoblasts.^37^ The patients who have a high risk of fracture are generally prescribed a combination of medications, one that grows the bone quickly and another that slows bone breakdown. This combination is found to be very effective.^38^

Despite having all these treatment processes, there are various limitations, such as the intake of bisphosphonates on a daily basis for a long period may cause serious problems in the body and unusual fractures of the thigh bones. This unusual effect of this drug has made the doctors concerned about the time limit for the prescription of this medication.^39^ Drugs like raloxifene only prevent bone fractures in specific body parts, and the intake of this drug carries a risk of side effects, such as a blood clot, in the future.^40^ Hormone replacement therapy (HRT) is no longer considered a first-line treatment for osteoporosis due to the need for long-term administration and its association with an increased risk of adverse effects, including cardiovascular disease and breast cancer.^41^ If a person stops taking denosumab, the rate of bone breakdown becomes much higher than the rate of bone breakdown before taking the drug. Furthermore, it also increases the chances of spinal fractures. So before prescribing denosumab, doctors need to plan the treatment process in relation to its after-effects.^42^ There are various types of treatment procedures available for the treatment of osteoporosis, but most of them have a lot of side effects, and some of them are very expensive (Table 1).^43^

**Table 1:** Comparison of common osteoporosis treatments based on their side effects and cost considerations

Treatment type	Side effects/disadvantages	Cost considerations	Mechanism	Efficacy	Potential plant-based adjunctive nutritional approach^a^	Ref.
Oral bisphosphonates	Gastrointestinal irritation, oesophageal irritation, and uncommon osteonecrosis of the jaw	Low-priced and widely available	Inhibits osteoclasts by triggering apoptosis	Reduces vertebral fractures by 40% to 70%	Magnesium-rich foods and soy protein provide adjunctive nutritional support to bone health	51
Intravenous bisphosphonates	Flu-like symptoms upon infusion and uncommon osteonecrosis of the jaw	Moderate cost	Inhibits osteoclasts by triggering apoptosis	Reduces hip fractures by around 40%	Calcium from plants	51 and 52
Denosumab (injection)	Injection site response, hypocalcemia, and uncommon atypical fractures	High cost and necessitates a visit to a healthcare facility	Blocks RANKL to prevent osteoclast development	Blocks RANKL, preventing osteoclast formation	Boron and zinc	53 and 54
Selective estrogen receptor modulators (SERMs)	Hot flashes and heightened risk of blood clots	Moderate cost	Estrogen agonist in bone and antagonist in breast	Reduces vertebral fractures by 30% to 50%	Phytoestrogens (soy)	51
Hormone replacement therapy (HRT)	Increased risk of breast cancer and cardiovascular events	Moderate to high costs	Mimics estrogen to balance remodeling	Prevents bone loss, but has limited long-term utility	Soy isoflavones	55
Teriparatide (anabolic)	Hypercalcemia and injection site responses	High cost and short treatment duration	Stimulates osteoblasts *via* the PTH receptor	Increases BMD by 9–13% and decreases fractures	Plant proteins like soy	51 and 52
Vertebroplasty/kyphoplasty	Risk of cement leakage and treatment difficulties	High cost and hospital-based	Stabilizes broken vertebrae using cement	Pain reduction by 70–90%; minimal BMD impact	N/A (surgical)	44 and 47
Hip replacement surgery	Surgical risks and prolonged recovery	Very expensive and specialist care	Replaces shattered joints	Restores function after fracture	N/A (surgical)	56

a These potential plant-based adjunctive micro and macro nutrients do not replace the standard pharmacotherapy.

In addition to pharmacological therapy, there are various surgical options for treating osteoporosis, particularly when fractures occur or conservative treatments fail. Vertebroplasty is a minimally invasive technique that injects bone cement (polymethyl methacrylate, PMMA) into a fractured vertebra to stabilize it and reduce pain. It is excellent for quick pain relief but poses hazards, such as cement leaking.^44^ Kyphoplasty is similar to vertebroplasty, with the exception that the treatment involves the inflation of a balloon within the fractured vertebra to reestablish its height prior to the filling of the void with bone cement. It improves spinal deformity and reduces pain more effectively, but at a relatively high cost.^45^ Cement-Augmented Screws are used in internal fixation to improve screw stability in osteoporotic bone. This approach lowers fixation failure but may cause difficulties in surrounding bones.^44^ Hip Replacement Surgery is required in cases of serious hip fractures that cannot heal adequately. It restores damaged bone and joint components, restoring function.^46^ Open Decompression and Fusion Surgery is a procedure used to straighten and stabilize the spine in cases of neurological problems or severe spinal instability caused by vertebral fractures.^47^ When non-surgical treatments fail, these surgical techniques are used to fix osteoporosis, relieve pain, restore function, and improve quality of life.^48^ Given the limitations and obstacles involved with conventional osteoporosis medications, researchers are actively seeking alternate options, with phytonutrients emerging as a promising field of study for skeletal health during aging and osteoporosis management.^49,50^

## 4. Why should we focus on phyto macro and micro nutrients

Plants synthesise phytonutrients (phytochemicals) as a means of defence against parasites and ultraviolet radiation. Fruits, vegetables, whole cereals, legumes, lentils, and tea are abundant sources of these compounds. Owing to their antioxidant, anti-inflammatory, and immune-enhancing properties, phytonutrients provide significant benefits to humans. They enhance cardiovascular and cognitive performance and may serve to prevent cancer and osteoporosis. The most prominent phytonutrients, including carotenoids, flavonoids, resveratrol, glucosinolates, and phytoestrogens, enhance eye health, reduce inflammation, and support hormonal equilibrium. Consuming a variety of vibrant, plant-based meals abundant in these compounds supports the promotion of health and the prevention of illness.^57^

**Fig. 2:**
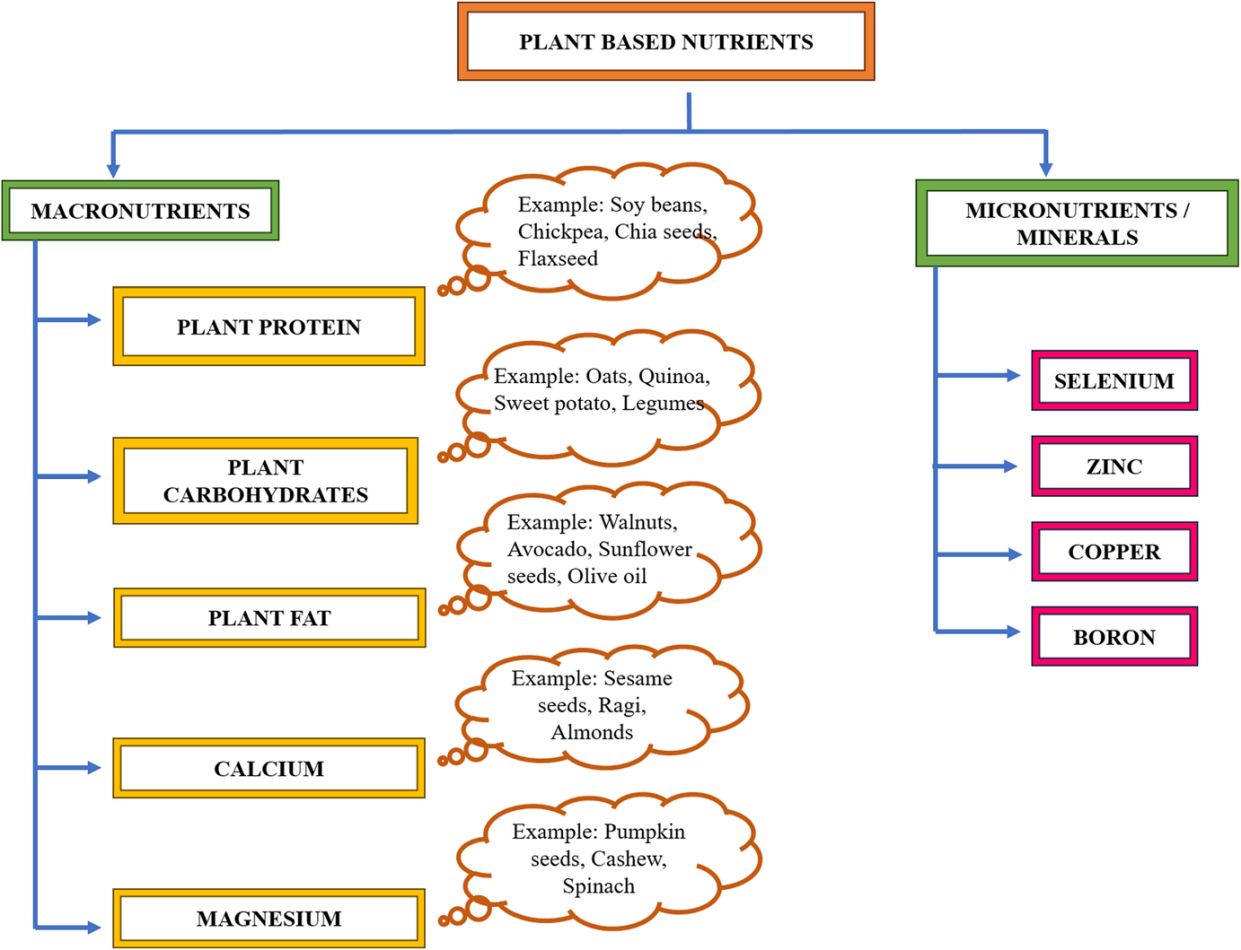
Classification of different nutrients and their natural sources.

Phytonutrients or nutraceuticals are generally classified into two types – macronutrients and micronutrients (Fig. 2). Phyto macronutrients include potassium, magnesium, calcium, and phosphorus, which play a major role in the overall growth and development of our bodies, along with maintaining the overall pH balance of our bodies. These phytonutrients help to make collagen, which is good for our bone health. The intake of these nutraceuticals on a daily basis saves our bones from getting fractures for any minor accidental reason (Fig. 2).^43,58^ Nutraceuticals, such as omega-3 and also some plant proteins, show anti-inflammatory effects on the body, it also reduces the activity of the osteoclast.^59^ Phyto micronutrients play a major role in maintaining a healthy life. These nutrients include plant-based estrogens and some minerals. One of the essential phytonutrients is plant-based estrogen, which has the ability to bind to the ERβ receptor, mimic the activity of estrogen, and initiate osteoblast differentiation.^60^ Vitamin K helps in the production of osteocalcin protein, which is very important for our bone health. Osteocalcin is primarily produced by osteoblasts, the bone-forming cells responsible for new bone synthesis.^61^ Consuming phytonutrients daily is much better than taking various medications that are used for the treatment of osteoporosis. Some of the reasons are as follows: fewer side effects, as these substances originate from natural sources, and they are good for our health as compared to the various prescribed medications available in the market. Additionally, they exhibit a variety of effects that are specifically designed to promote bone health, including anti-inflammatory properties. They are also cost-effective and can be consumed for an extended period.^43^

### 4.1 Different plant-based macronutrients for osteoporosis treatment

#### 4.1.1 Plant proteins and peptides

Proteins are the building blocks of our body. Plant-derived proteins are crucial structural macromolecules for the development and maintenance of the collagenous bones and are also involved in osteogenic signaling pathways.^62^ In addition to their nutritional advantages, the osteoprotective effects of plant proteins are affected by the structure–activity correlations of their bioactive peptide molecules. During gastrointestinal metabolism, plant proteins undergo breakdown into low-molecular-weight peptides, and their biological activities are influenced by amino acid content, sequence order, and physicochemical characteristics.^63^ Peptides rich in acidic amino acids, such as aspartic acid and glutamic acid, have negatively charged carboxyl groups that may coordinate with calcium ions *via* electrostatic interactions. These calcium-binding peptides may improve calcium solubility and promote intestinal absorption, thereby aiding bone synthesis and bone health.^64^

Soy protein is remarkable among plant protein sources since it provides necessary amino acids and acts as a natural transporter of bioactive isoflavones. The osteoprotective efficacy of these isoflavones is intricately linked to their chemical composition.^65^ Compounds such as genistein and daidzein have a diphenolic aromatic structure that closely resembles that of natural 17β-estradiol. The spatial configuration of hydroxyl groups on the aromatic rings facilitates hydrogen-bond interactions with amino acid residues in the ligand-binding domain of estrogen receptors, namely estrogen receptor-β (ER-β).^66^ Particularly during the menopausal transition, these interactions allow isoflavones to have estrogen-like effects on bone tissue, encouraging osteoblast development while reducing osteoclast-mediated bone resorption. Therefore, bioactive peptides and related isoflavones may work in concert to preserve bone homeostasis and prevent osteoporosis.^67^ Several studies have shown that soy protein helps make new bones by increasing the activity of osteoblasts and decreasing the activity of osteoclasts.^68^

Recent research has shifted its attention beyond soy proteins and isoflavones to explore novel cyclic plant-derived peptides, specifically flaxseed cyclolinopeptides (CLPs), as potential regulators of bone remodeling. In contrast to traditional linear peptides, cyclic peptides exhibit a closed-ring molecular structure that has the potential to boost protection against enzymatic degradation and increase biological stability. These structural features have generated considerable attention regarding their potential therapeutic uses.^69,70^ At the molecular level, it has been observed that cyclolinopeptides demonstrate both osteogenic and anti-resorptive effects. Specific CLPs, such as CLP-A, CLP-E, and CLP-P, have demonstrated the ability to activate the PI3K/Akt signaling pathway in osteoprogenitor cells. This activation results in an increase in alkaline phosphatase (ALP) activity and promotes the enhanced differentiation of osteoblasts.^71^ Furthermore, cyclolinopeptide J demonstrates anti-osteoclastogenic activities through the modulation of osteogenic RUNX2 biomarkers, achieved by the activation of the Wnt/β-catenin and BMP/Smad signaling pathways.^72^ CLPs facilitate the removal of phosphate groups and the movement of β-catenin into the nucleus, which in turn increases the expression of genes associated with osteoblasts. At the same time, they reduce the activity of the RANK/RANKL signaling pathway, thereby inhibiting the formation of osteoclasts. The PI3K-Akt and Ras pathways play a significant role in the process of osteogenesis mediated by CLP. Flaxseed cyclolinopeptides, through their coordinated regulation of osteoblastogenesis and osteoclastogenesis, emerge as a promising group of plant-based bioactive peptides.^71,73^ They hold potential for maintaining bone homeostasis and addressing the progression of osteoporosis. A study revealed that chitosan-based bone-targeted nanoparticles incorporating CLP result in a remarkable 13.6-fold increase in bone accumulation. These nanoparticles synergistically release Ca^2+^, which further enhances bone formation, as well as restores trabecular architecture in ovariectomized mice. This demonstrates their dual capabilities of promoting bone formation and inhibiting bone resorption.^72^

#### 4.1.2 Plant carbohydrates

There are various types of plants that provide different forms of complex carbohydrates, such as oligosaccharides and inulin. The different forms of complex carbohydrates are found in plants, such as legumes and green bananas. These complex carbohydrates contain fibres that help to break down the good bacteria that are already present in the large intestine. This breakdown process leads to the formation of short-chain fatty acids (*e.g.* butyrate). The short-chain fatty acid creates an acidic environment that helps to dissolve calcium and magnesium for better absorption. Calcium and magnesium are very important for our bone health.^74^ Butyrate plays a major role in our bone health as it helps to develop bone-building cells, which are osteoblasts, and it also inhibits the formation of osteoclasts, which are the bone-breaking cells. Butyrate also helps to absorb the minerals that help in the growth and development of the bones.^75^ People have been taking inulin and oligosaccharides for a long period. It is found in various sources that are easily available, such as wheat, onions, and garlic. From the record, it has been found that in North America, people consume almost ∼4 grams of oligosaccharides and inulin on average, but in Western Europe, people consume almost ∼10 grams of oligosaccharides and inulin on average.^76^

#### 4.1.3 Plant fats

Alpha linoleic acid (ALA) is a type of fat that is found in various sources, such as chia seed, walnuts, and flaxseed. It inhibits the inflammatory signals that destroy bones while encouraging the activity of osteoblasts. As it reduces the signal, the action of bone breakdown slows down. It increases the production of bone morphogenetic proteins, which increase the signals of osteoblasts, which increases the formation process of new bones.^59,77^ From various research, it has been found that the intake of ALA on a daily basis makes our bones much denser and stronger by increasing the absorption of minerals. Further studies showed that the intake of omega-3, that is ALA, on a daily basis inhibits fractures on minor falls and makes the bones very strong.^78^ The intake of phytonutrients on a daily basis makes our bones strong as it balances the level of hormones, causes anti-inflammatory action, and increases the absorption of minerals. This is how it helps in the formation of a stronger skeletal system.

#### 4.1.4 Calcium

**Fig. 3:**
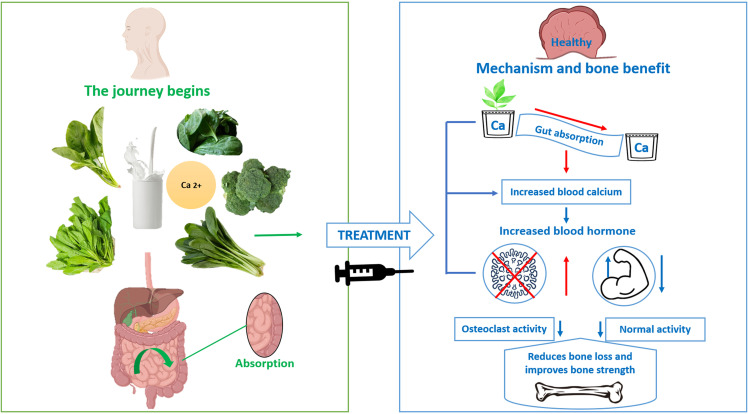
Working mechanism of calcium as a phytonutrient.

Calcium is recognized as a crucial component for bone health. Calcium comprises hydroxyapatite, a compound that facilitates bone formation and enhances their strength.^43^ In our daily diet, enough calcium should be present to maintain the levels of parathyroid hormones. If the amount of parathyroid hormones becomes high, it causes the bone to break down much faster, so it is very important to maintain the amount of calcium in our diet to maintain good bone health.^79^ According to some studies, it has been found that the intake of vitamin D, along with almost 1200 mg of calcium per day, makes our bones strong, maintains our bone health and also helps to prevent bone breakdown.^80^ Calcium supplements are very effective, but intake on a daily basis may cause heart disorders. Thus, to avoid various disorders, the intake of plant-based calcium is much safer. Beans and leafy greens are rich in calcium. Importantly, botanical calcium bioavailability relies totally on the dietary matrix. Phytates and oxalates are present in high concentrations in certain leafy vegetables, including Swiss chard and spinach. These anti-nutritional substances generate insoluble calcium oxalate and calcium phytate complexes with free calcium ions in the gut, preventing intestinal absorption.^81,82^ The fractional absorption of plant-derived calcium exhibits a strict opposite relationship with the specific oxalate content present in the plants. In high-oxalate plants, the molar ratio of oxalate is in excess relative to calcium, which nearly completely precipitates the mineral into insoluble complexes and results in functional absorption rates below 5%. Thus, these plants are rich in essential calcium yet have limited functional bioavailability.^83^ Medium-oxalate meals (*e.g.*, certain beans and legumes) bind a part of the mineral, resulting in moderate absorption. In contrast, cruciferous vegetables, such as kale, bok choy, and broccoli, are low in oxalates and do not possess this metabolic barrier. Due to inadequate oxalate to bind the mineral, calcium predominantly exists in its free and ionized form within the gastrointestinal lumen, leading to an exceptionally high fractional absorption rate (often 40–50%), which renders them significantly superior substrates for osteoblast-mediated bone mineralization (Fig. 3).^83–85^ There are several mechanisms through which calcium treats osteoporosis. Mineralization of bone and formation of hydroxyapatite: the formation of hydroxyapatite crystals (Ca_10_(PO_4_)_6_(OH)_2_), which give bone its inorganic hardness and compressive strength, requires calcium. Weak bones result from poor mineralization caused by insufficient calcium. Regulation of Parathyroid Hormones (PTHs): low dietary calcium causes a dip in serum calcium, which in turn causes an increase in PTH release. To restore serum calcium, PTH increases osteoclast activation (bone resorption). The increase in PTH over time causes net bone loss. Consuming enough calcium lowers PTH release, which in turn lowers bone resorption.^50^ Cooperation with other nutrients and vitamin D: active vitamin D (calcitriol) is essential for the gut's absorption of calcium. Even a high calcium intake may not be well absorbed without vitamin D. The incorporation of calcium into bone is also influenced by other minerals, such as magnesium and vitamin K. According to reviews, taking calcium supplements along with vitamin D improves BMD and lowers the risk of fractures more than taking calcium alone.^86^ Preventing fractures and bone loss: according to clinical studies and meta-analyses, postmenopausal women who take calcium supplements (typically in addition to vitamin D) had a relatively low rate of bone mineral density loss and a low risk of hip and vertebral fractures. Those with low baseline intake or insufficiency get a more noticeable benefit.^87^

#### 4.1.5 Magnesium

Magnesium is an essential biological cofactor for more than 300 enzymatic activities, acting as a primary structural element of the skeletal system and a vital regulator of bone homeostasis. Magnesium structurally governs the solid-state stability of hydroxyapatite, the optimal magnesium concentrations inhibit the development of excessively large hydroxyapatite crystals, thereby maintaining both the mechanical strength and quality of the structure of the bone matrix. Moreover, magnesium serves as a crucial catalyst for alkaline phosphatase (ALP) activity, an important enzyme for the proper deposits of minerals in the bone matrix.^88,89^ A deficiency of magnesium has an adverse influence on the body. It inhibits the osteoblast process, which is responsible for bone regeneration, while accelerating the osteoclast process, which involves bone resorption. A deficiency of magnesium also increases inflammatory levels, adversely affecting bone health by destabilizing and weakening it. Records indicate that adequate daily magnesium consumption by women throughout menopause has beneficial outcomes, since it reduces bone resorption and enhances bone strength. Magnesium is fundamentally involved in the endocrine control of bone mass. It is biochemically essential for the hepatic and renal hydroxylation processes that activate vitamin D, as well as for the proper synthesis and release of parathyroid hormone (PTH). As a result, magnesium shortage significantly disturbs this endocrine axis, causing reduced calcium absorption and a paradoxical decrease in PTH effectiveness, which together expedite pathological bone resorption.^90,91^

**Fig. 4:**
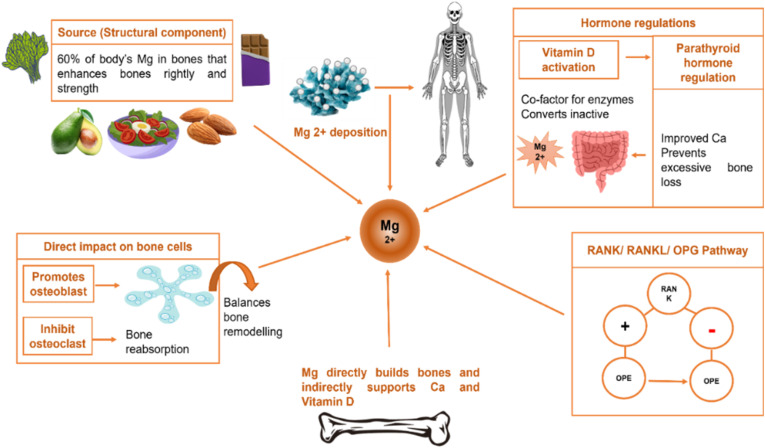
Working mechanism of magnesium as a phytonutrient.

The coupling of bone remodeling is fundamentally disrupted by magnesium deficiency, which alters the RANKL/OPG signaling axis. The RANKL/OPG ratio is substantially increased by low intracellular magnesium, which serves as a potent molecular signal that accelerates osteoclastogenesis while at the same time suppressing Wnt-mediated osteoblast activity. In addition to its direct structural and endocrine functions, magnesium plays a significant role in bone remodeling by suppressing upstream inflammatory pathways (Fig. 4).^92,93^ A deficiency in magnesium results in systemic oxidative stress and a significant increase in the expression of osteoclastogenic cytokines, such as TNF-α and IL-1β. Intracellular magnesium functions as a physiological calcium antagonist. It prevents the pathological intracellular calcium influxes that initiate the generation of reactive oxygen species (ROS) by modulating calcium-permeable channels. Adequate magnesium deprives the NF-κB signaling cascade of its inflammatory stimuli, thereby avoiding the downstream transcription of bone-resorbing cytokines, as ROS is the primary activator of this pathway. In medical applications, it has been demonstrated that the skeletal matrix is structurally fortified against osteoporotic fractures by maintaining an adequate daily magnesium supplement during the menopausal transition, which effectively mitigates osteoclast-mediated bone disintegration.^50,92,93^

### 4.2 Different plant-based micronutrients

#### 4.2.1 Minerals

There are various minerals that are very important for our bone health. For maintaining bone health and to avoid fractures even on minor falls, we need to consume a proper amount of minerals on a daily basis. There are various types of minerals, including zinc, boron, copper, and selenium. Our bones undergo continuous growth and remodelling every day.^94^

##### 4.2.1.1 Zinc

Zinc is a trace mineral that is essential for bone health. It is a potent regulator of bone homeostasis, producing a dual-action therapeutic effect. Specifically, it stimulates osteoblast-mediated bone formation and also prohibits the signals from NF-kB that stimulate osteoclast-mediated bone resorption. Zinc is a structural requirement for the enzymatic synthesis of the collagenous bone matrix, which is essential for maintaining biomechanical strength. There are two different mechanisms through which zinc treats osteoporosis.^95^ The initial process is stem cell differentiation, during which zinc enters hBMSCs (human bone marrow-derived mesenchymal stem cells) and induces a significant rise in the level of cAMP (cyclic adenosine monophosphate). The PKA (protein kinase A) is activated by an increase in cAMP levels. The cAMP/PKA signaling cascade concludes with the induction and phosphorylation of critical osteogenic transcription factors, including CREB (cAMP response element-binding protein) and RUNX2.^96^ These two genes are crucial for bone formation. The master regulators direct osteoblasts to form new bones. CREB and RUNX2 activation turns hBMSCs into osteoblasts.^96^

Zinc's antiresorptive capacity relies on its profound disruption of the NF-κB signaling pathway. In inflammatory microenvironments, hyperactivated NF-κB is a primary driver of osteoclastogenesis and simultaneously acts as a direct inhibitor of osteoblast function. Zinc interferes with the NF-kB pathway and inhibits the activation of the NF-kB protein inside the cell, thereby inhibiting the activity of NF-kB. This blockade prevents the degradation of IκBα, securely sequestering NF-κB in the cytoplasm and preventing its nuclear translocation.^97^

##### 4.2.1.2 Copper

**Fig. 5:**
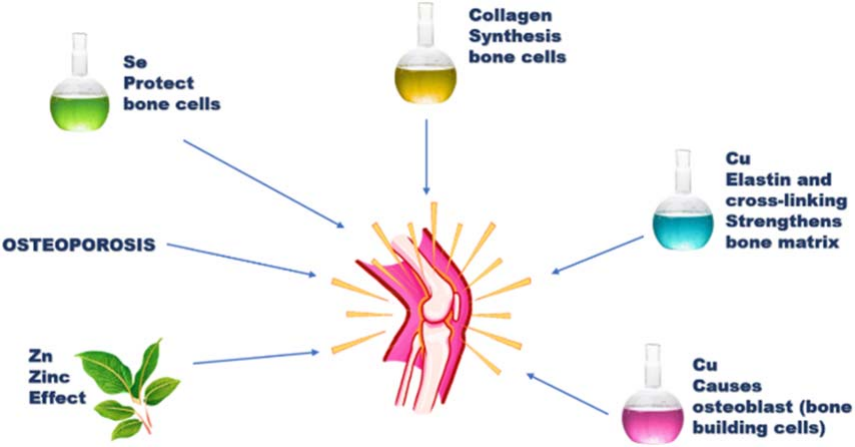
Different minerals as phytonutrients for the treatment of osteoporosis.

Copper is one of the most essential nutrients because it helps the lysyl oxidase enzyme to hold the main building block fibers of our bone, that is, the elastin and collagen, and makes the bone strong.^98^ There are various mechanisms through which osteoporosis can be treated using copper. Collagen and bone matrix stability *via* cross-linking by lysyl oxidase (LOX) requires copper to oxidatively deaminate lysine and hydroxylysine residues in collagen and elastin, facilitating the formation of cross-links that provide tensile strength to bones (Fig. 5). A deficiency of copper diminishes LOX activity, thereby compromising bone and collagen integrity.^99^ Additionally, copper is a crucial component for detoxifying reactive oxygen species (ROS) that can harm osteoblasts and encourage osteoclastogenesis. Redox balance preservation aids in preserving cell viability and bone matrix.^50^ At the right dosage, copper ions can stimulate osteoblast development by upregulating osteogenic genes (such as RUNX2, ALP, osteocalcin, and collagen type I) and encouraging osteoblast proliferation. In biomaterial scaffold models, copper-doped biomaterials have demonstrated increased osteogenesis and angiogenesis.^100^ The formation of blood vessels is essential for bone regeneration and healing. Copper promotes vascular ingrowth and supplies nutrients and osteoprogenitor cells by enhancing the production of angiogenic factors, including VEGF. For example, scaffold and MSC studies indicate that copper in scaffolds promotes angiogenesis.^101^

##### 4.2.1.3 Boron

Boron is an important mineral that increases bone density by facilitating the binding of structural building blocks, such as calcium and magnesium, as well as regulating hormone levels (such as estrogen and progesterone), which are critical for bone health in old age.^102^ Boron helps treat osteoporosis by enhancing bone mineral absorption and retention (Ca, P, and Mg), particularly when paired with estrogen, which results in significantly higher mineral retention than estrogen alone.^103^ Boron, at low concentrations, stimulates osteoblast activity *in vitro* by accelerating mineral deposition, encouraging cell differentiation in the osteogenic lineage, boosting ALP (alkaline phosphatase), and upregulating bone-formation genes and proteins, such as RUNX2 and BMPs.^104^ Boron inhibits bone resorption and osteoclastogenesis by lowering the number of active osteoclasts and TRAP activity (an osteoclast marker) in animal models of osteoporosis and alveolar bone loss.^105^ Boron also facilitates hormonal regulation by raising vitamin D consumption and serum testosterone and estradiol levels, both of which are important for bone health maintenance, particularly after menopause.^106^ Boron may also modulate antioxidant and anti-inflammatory pathways, thereby reducing oxidative stress and inflammatory cytokines, which would otherwise increase osteoclast activity and bone loss.^107^

##### 4.2.1.4 Selenium

Selenium is an important mineral for bone health because it increases glutathione peroxidase activity, which helps lessen the detrimental effects of reactive oxygen species (ROS) on cells, such as osteoblasts, slowing the bone breakdown process and strengthening bones.^108^ As a result, integrating sufficient phytonutrients or nutraceuticals into the daily diet strengthens bones and lowers the risk of osteoporosis (Fig. 5).^43^ Selenium primarily helps to manage osteoporosis through antioxidant defense, since it produces selenoproteins (GPx and TrxR) that scavenge ROS, lowering oxidative stress in bone cells and maintaining osteogenic differentiation while inhibiting osteoclast activation.^109^ Selenoprotein W (SELENOW) is required for appropriate osteoclast maturation through RANKL-dependent regulation. Consequently, selenoprotein dysregulation has a direct impact on bone resorption.^110^ Selenium protects osteoblast function by inhibiting oxidative stress and altering signaling cascades (such as ERK), thereby sustaining ALP activity, collagen synthesis, and mineralization.^111^ Selenium also indirectly regulates bone remodelling by decreasing inflammation (by oxidative stress management) and detoxifying heavy metals.^109^

It has been revealed that these macro and micronutrients have a possible influence on osteoblasts and osteoclasts. Various researchers are developing unique formulation techniques to demonstrate the potential of these phytonutrients for the regeneration of bone structures. There are several examples of studies that have been conducted on this topic in the later section.

## 5. Formulations based on phytonutrients for osteoporosis

The molecular effectiveness of plant-derived macro- and micronutrients in bone remodeling is well-established. However, their clinical efficacy is limited by pharmacokinetic challenges when provided as standard oral supplements. Various phytochemicals, especially polyphenols and flavonoids, are notably defined by their low water solubility, fast systemic clearance, and significant presystemic hepatic first-pass metabolism.^112,113^ Innovative drug-delivery formulations and methodologies, such as tailored micro/nanocarriers and scaffolds, could be employed to avoid these biological limitations. These innovative delivery technologies fundamentally transform the pharmacokinetic profile and significantly enhance the bioavailability of nutrient absorption.^114^ Consequently, novel delivery systems may overcome pharmacokinetic barriers and enhance the therapeutic efficacy of certain plant-derived biological compounds in the therapy of osteoporosis.

### 5.1 Macronutrients

**Fig. 6:**
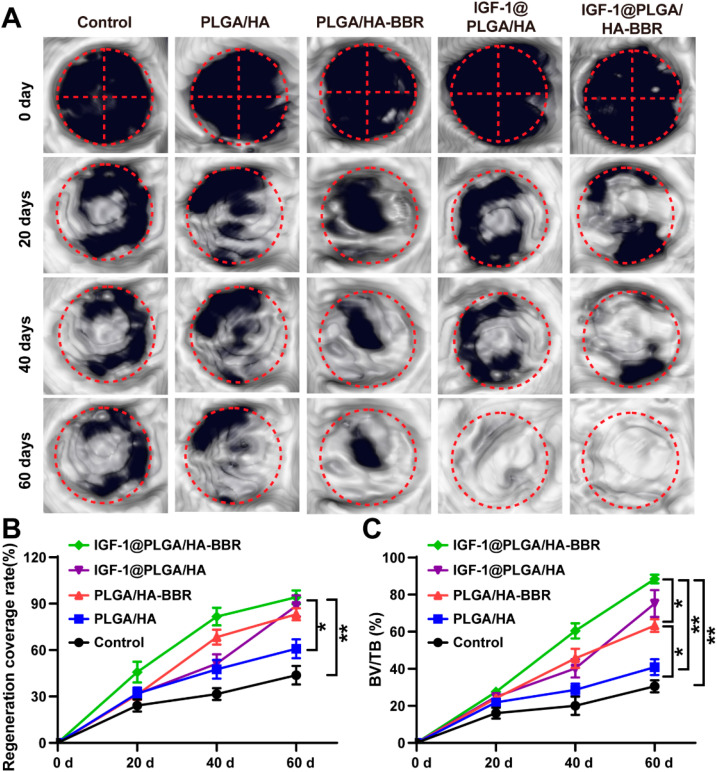
(A) Microspheres' repair effect on skull injury. 3D micro-CT images showing bone regeneration from various samples at different time points in the rat calvarial bone defect region. A red dashed circle with a 5 mm diameter marks the defect region. (B) Quantification analyses of the skull defect regeneration coverage rate in each group. (C) Quantification analyses of bone volume over total volume (BV/TV) following skull injury repair using various microsphere sets [figure adopted from Chen *et al.*, 2023;^115^ Licence under CC BY 4.0].

Various researchers have conducted studies on phyto-macronutrients for bone tissue engineering. These studies demonstrate the potential of multiple macronutrients to generate bone tissue and reduce osteoclast activity (Table 2). For instance, Chen *et al.* developed a bone tissue engineering method that employs phase separation and electrified liquid jets to fabricate an IGF-1@PLGA/HA-BBR composite microsphere formulation. The materials consist of inorganic hydroxyapatite (HA) and polymer PLGA, which have been surface-modified with IGF-1 (growth factor) and loaded with berberine (BBR). All microsphere groups demonstrated excellent sphericity, with particle sizes ranging from 300 to 450 µm. Characterisation revealed strong synergistic effects, high biocompatibility, and sustained release of both BBR and IGF-1. In particular, the IGF-1R/PI3K/AKT/mTOR pathway is co-activated by BBR and IGF-1, thereby accelerating cell mineralisation and promoting osteogenic differentiation (ALP assay) on MC3T3-E1 cells. They found that the microspheres established a complete bony connection in rat calvarial defect healing (Fig. 6); the study indicates that the microspheres are highly effective and show tremendous promise.^115^

**Table 2:** Empirical evidence of nutrient-derived formulations for osteoporosis: types of formulations, research models, and therapeutic outcomes

Micronutrient/macronutrient	Type of formulation	Property of formulation	*In vitro*/*in vivo* study cell line/species	Research outcome	Ref.
Vitamin C and vitamin K (micronutrient) from quercetin	Phytosome nanoparticles	Particle size: 70 ± 7.44 nm; encapsulation efficacy: 98.4%	Adult female albino rats; age: 10–13 weeks; weight: 120–150 g	Exposure to a low dose of free quercetin (10 mg kg^−1^) produced no noticeable effects since it was not properly absorbed for a short period, while QP (quercetin-loaded phytosome) at the same dose drastically reduced the risk of bone loss	125
Vitamin C and vitamin K (micronutrient) from quercetin	Porous calcium-deficient hydroxyapatite (CDHA) scaffold	Compressive strength: 2 ± 1.8 MPa	Pre-osteoblast cell (MC3T3-E1), osteoclast cell (RANK-treated RAW 264.7)	The QC-CDHA (quercetin-loaded calcium-deficient hydroxyapatite) scaffolds greatly enhanced the proliferation, differentiation and mineralization of pre-osteoblast cells (MC3T3-E1), while the progression and differentiation of osteoclast cells (RANK-treated RAW 264.7) were substantially repressed	126
Vitamin A (micronutrient)	Liposome	Size: 151 d nm; zeta potential: −13.7 mV	SaOS-2 cells	After a period of 72 hours, osteoblast cells treated with vitamin A liposomes generated a 45–50% increase in SaOS-2 cell proliferation in comparison to those with free vitamin A	127
Vitamin D_3_ (micronutrient), calcium (macronutrient)	Microparticles	Size: 1.33 ± 0.35 µm	Kunming mice (female, age: 6–8 months) and Sprague-Dawley rats (female, weight: 180–220 g)	In contrast to VD_3_@CHP (vitamin D_3_/calcium hydrophosphate microparticles), VD_3_@HAP (vitamin D_3_/hydroxyapatite microparticles) exhibited enhanced calcium absorption, lessened marrow cavity area, as well as improved osteoporosis symptoms in the rat model	128
Vitamin K_2_ (menaquinone-7), strontium citrate and fructoborate (micronutrient)	Tablets	Drug release: >95% within 45 minutes	Female Sprague-Dawley rats (age: 12 weeks; weight: 220 ± 20 g)	When compared to vehicle controls, the administration of 100 mg kg^−1^ of OMC (osteotropic micronutrient complex) substantially increased femoral bone mineral density by 28.7%	129
Soybeans (macronutrient)	Gold nanoparticles	Size: 22–69 nm	HUVEC cells; male rats	After gold nanoparticle administration, the serum levels of bone mineral content markers increased, whereas urine and serum levels of bone resorption markers lowered. Particularly with gold nanoparticles, a rise in tibia and femur strength was found	130
Vitamin C from rutin as an antioxidant, saponin (micronutrients)	Nanoparticles	Size: 139.6 ± 1.55 nm; polydispersity index: 0.124 ± 0.01; zeta potential: 12.2 ± 0.91 mV	*In silico* study: molecular docking analyses, molecular dynamics simulations, and evaluation of drug-likeness properties. Cell line: mouse fibroblast cell line (L929)	GEE-loaded CNPs (chitosan nanoparticles) showed no cytotoxic effect at relevant concentrations, while this nanoformulation was less hazardous than GEE (*Gypsophila eriocalyx* extract)	131
Vitamin C from rutin as an antioxidant (micronutrients)	Nanosuspension	Particle size: 122.85 ± 5.02 nm	*In situ* study: single-pass intestinal perfusion (SPIP) study; *in vitro* study: MG-63 cell line. *In vivo* study: female Wistar rats; weight: 190–220 g	After oral administration in a rat model, the results showed a four-fold rise in the RUT-NS's (rutin nanosuspension) plasma concentration maxima (*C*_max_) and area under the curve (AUC0–24 h) compared to the RUT	132
Zinc (micronutrient)	Nanoparticles	Particle diameter: 756 nm	Four-week-old female Sprague Dawley (SD) rats (65–75 g)	In contrast to the untreated or ZnSO_4_-treated groups, the group treated with zinc-containing β-tricalcium phosphate (ZnTCP) nanoparticles produced a substantially higher bone mineral content (BMC) of jaw bone. The ZnTCP-treated group possessed significantly higher body weight, femur BMC, and bone mechanical strength (BMS)	133
Calcium (macronutrient)	Nanocomposite	Particle size: less than 400 nm	Eight-week-old female Sprague-Dawley rats; RAW 264.7 cells	Tartrate-resistant acid phosphatase (TRAP)-positive cells dropped because of the calcium salt nanocomposites' suppression of osteoclastic activity, including RANKL expression. The calcium salt nanocomposites significantly enhanced bone volume and density, increased osteoblasts, and reduced osteoclasts after being administered orally to osteoporotic rats throughout 45 days, in contrast to those in control rats that were not given any treatment	134
Micronutrient: boron (boron nitride); macronutrient: calcium and phosphorus (hydroxyapatite)	Scaffolds	Pore diameters: 100 µm in large pores to 30 µm in small pores	Female Sprague Dawley rats; weight: 230–250 g	In the first and second week following the procedure, osteoporotic rats exhibited no signs of recovery. The groups were treated with poly-(lactide-*co*-glycolide) (PLGA) scaffolds containing 10% HA (hydroxyapatite), 2.5% BN (boron nitride) + 10% HA, 2.5% BN and 5% BN; specifically, the 2.5% BN + 10% HA group exhibited healing in the fourth week	135
Zinc (micronutrient)	Nanoparticles	Particle size: 14.74 and 18.08 nm	Wistar rats; age: 12 weeks	Risedronate/zinc-hydroxyapatite (RIS/ZnHA) nanoparticles have been observed to deliver therapeutic advantages over the RIS or RIS/HA therapy in the prevention and management of postmenopausal osteoporosis following an *in vivo* study in rat models	136
Zinc (micronutrient)	Nanoparticles	Average length: 64 ± 10 nm	Female Sprague–Dawley rats weighing 200–250 g	While it is related to reducing excessive bone turnover and preventing osteoporosis, the combination therapy of OVX/E2 + ZnHA was much more effective than the individual treatments	137
Zinc (micronutrient)	Microparticles	Average size: 4 µm	12-week-old male Wistar rats	Following *in vivo* investigation, the bone mass resulting from Sclmab-loaded hydroxyapatite/chondroitin sulfate composite (HAp/ChS) microparticles containing zinc (Zn) cations was substantially greater than that of HAp/ChS microparticles alone in 1% of the hazard ratio (HR); Sclmab-loaded HAp/ChS microparticles containing no Zn cations were not significantly different from non-Sclmab-loaded HAp/ChS microparticles	138
Zinc (micronutrient)	Suspension	—	Four-week-old female Sprague Dawley (SD) rats (average body weight: 65–75 g)	During 12 weeks, the C8Zn (zinc octanoate) and C18Zn (zinc stearate) groups' bone mineral content (BMC) and femur's bone mechanical strength (BMS) were considerably higher than the control group; however, the ZnSO4 (zinc sulfate hepta-hydrate) group's findings remained unchanged	139
Vitamin D_3_ (micronutrient)	Nanocochleates (nanocochs)	Particle size: 319.4 ± 27.3 nm; polydispersity index: 0.38 ± 0.08	Male Wistar rats; age: 6–7 months; weight: 210 ± 30 g	In rats, oral nanococh-D_3_ improved osteogenic biomarkers, boosted bone mechanical strength, and greatly increased the bioavailability of vitamin D_3_. In treated rats, this formulation greatly lowered RANK and RANKL gene expression. TRAP staining revealed an enormous decrease in osteoclasts using nanococh-D_3_ in osteoporotic rats, whereas histomorphometric analysis indicated significant repairs in bone tissues	140
Zinc (micronutrient); calcium (macronutrient)	Nano-powdered particles	Particle size: 389 ± 30.4 nm	7-week-old Sprague Dawley rats	In rat models, Zn-NPOS (zinc-activated nanopowdered oyster shell) treatment exhibited better trabecular architecture, along with improved bone strength, than NPOS and POS treatments	141
Zinc and silicon (micronutrient)	Electroconductive biocomposite	Surface electroconductivity: 5625 S m^−1^	Human umbilical vein endothelial cells (HUVECs), RAW264.7 cells	Reduced graphene oxide/zinc silicate/calcium silicate (RGO/ZS/CS) conductive biocomposite extracts could hinder RAW264.7 cells from transforming into osteoclasts	142
Calcium (macronutrient)	Nanocapsules	Diameter of particles: 200 nm	MLC-6 cells	Mice exposed to CaP-DeCA/Sim (calcium phosphate-simvastatin/deoxycholate assembly) exhibited a significant improvement in bone mineral content (BMC) in both the injected as well as uninjected sites	143

Another type of macronutrient, such as proteins, is also studied. Wu *et al.* address significant bone defects, and this study presents a novel organic–inorganic composite scaffold (EHSS/HAp) made of hydroxyethyl cellulose (HEC), soy protein isolate (SPI), and hydroxyapatite (HAp). The scaffold, which was made by lyophilization and biomimetic mineralization, has better mechanical properties, an interconnected porous structure, and a ratio (1.65 at 70% SPI) that is very similar to that of genuine bone (1.67). Excellent cytocompatibility and the capacity to significantly increase osteogenic gene expression (Col-1, RUNX2, OPN, and OCN) in MC3T3-E1 cells were validated *in vitro*. Importantly, the scaffold with 70% SPI significantly encouraged new bone formation when applied to rats with critical-sized cranial defects, resulting in nearly full defect occupation at 12 weeks and showing great promise for bone regeneration.^116^ Similarly, Arjmandi *et al.* looked at how soy protein, a phyto macronutrient high in isoflavones (phyto micronutrients), affected bone metabolism in postmenopausal women who were either on or off hormone replacement therapy. In a three-month double-blind research, seventy-one women were given either soy protein (SP) or milk-based protein (MBP) at 40 g per day. Both boosted serum IGF-I, a bone formation marker, although SP had a larger effect. SP significantly decreased urine deoxypyridinoline, a bone resorption marker, whereas MBP did not. Women on MBP had higher urine calcium loss than SP. In non-HRT women, SP increased IGF-I by 97%. Thus, soy protein and isoflavones promote bone health and calcium balance, lowering the risk of osteoporosis.^117^ Hannan *et al.* looked at the association between protein intake and bone mineral density (BMD) in older men and women. Protein was the primary macronutrient in question, derived primarily from dietary and animal sources. Although no formulation was created, the data highlighted the possibility of developing a protein-based nutritional supplement for osteoporosis prevention. A formulation could include protein isolates, such as whey or soy protein, calcium, vitamin D_3_, and magnesium to improve bone strength. The study revealed that an appropriate protein diet promotes bone density and decreases bone loss in the elderly.^118^

In another study on fatty macronutrients, EI Wakf *et al.* developed a food formulation containing soybean oil (15% w/w) or sesame oil (10% w/w) to test its osteoprotective effects in ovariectomized rats. These oils, which contain essential fatty acids and hence qualify as phyto-macronutrients, were the only active ingredients mentioned in the formulation. Supplementation improved serum and bone calcium and phosphorus levels, increased antioxidant enzyme activity (SOD, CAT, and GSH), decreased oxidative stress markers (MDA and PC) and inflammatory mediators (TNF-α, CRP, and ACP), and normalized WBC numbers. Overall, the data indicated that soybean and sesame oil-rich diets can help prevent bone loss caused by estrogen deprivation.^119^

**Fig. 7:**
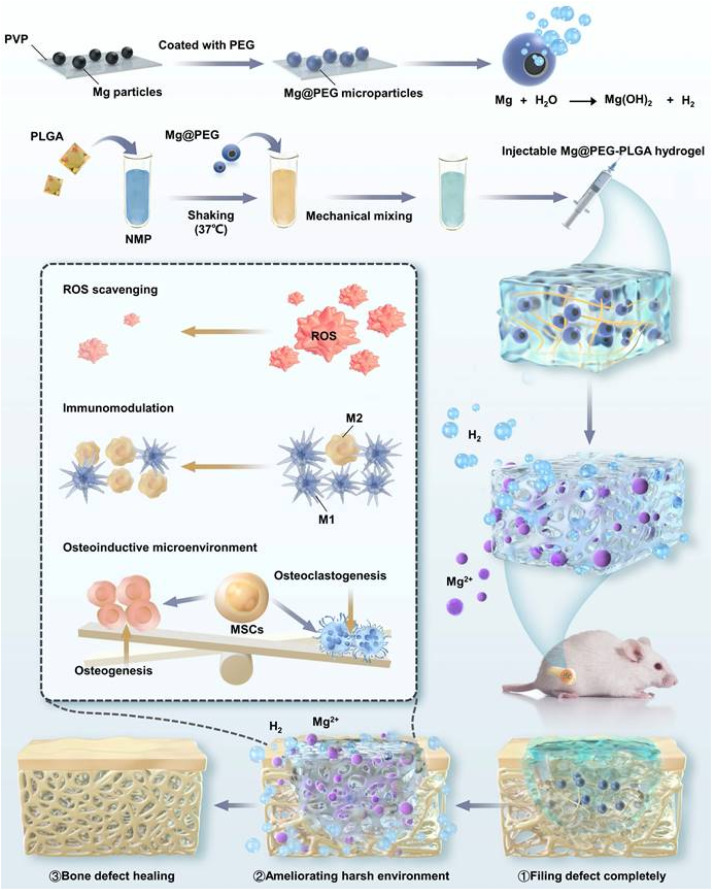
Schematic of the mechanism by which the injectable Mg@PEG-PLGA hydrogel effectively promoted osteoporotic bone regeneration [figure adopted from Zhou *et al.*, 2024;^121^ Licence Under CC BY 4.0].

Ryder *et al.* showed that magnesium is an important element for bone growth and calcium metabolism. In this study, magnesium supplementation was made up of oral tablets containing magnesium oxide or citrate as the active ingredient and excipients, such as microcrystalline cellulose, magnesium stearate, and starch. Optional additions, such as calcium carbonate and vitamin D_3_, were added to improve bone health. Post-formulation evaluations included physicochemical tests, assays, content uniformity testing, dissolution testing, and stability investigations. The findings revealed that high magnesium intake was substantially associated with high bone mineral density (BMD) in older white men and women, but not in black participants. The data imply that magnesium supplementation may assist elderly people in retaining bone strength and lower their risk of osteoporosis.^120^ Zhou *et al.* used magnesium (Mg) as the principal medicinal ingredient. Researchers created an injectable hydrogen-releasing Mg@PEG-PLGA hydrogel scaffold composed of magnesium, polyethylene glycol (PEG), and poly(lactic-*co*-glycolic acid) (PLGA). The tailored 2Mg@PEG-PLGA hydrogel efficiently lowered ROS levels, regulated inflammation by boosting M2 macrophage polarization, inhibited osteoclastogenesis, and stimulated osteogenesis, resulting in fast osteoporotic bone defect repair (Fig. 7). Overall, the findings imply that H_2_-releasing magnesium-based hydrogels have high potential as less-invasive implants for the improved regeneration of osteoporotic bone defects.^121^

Yang *et al.* formulated calcium yak caseinate (CYC), a calcium-enriched yak milk casein protein formulation, and administered it to ovariectomized rats for 6 weeks to prevent postmenopausal osteoporosis. CYC predominantly uses caseinate protein as a phyto/macronutrient source, along with calcium. The study found that CYC improved bone physical indices and maintained trabecular structure, and, most importantly, low-dose CYC prevented bone mineral-density loss by lowering serum calcium, alkaline phosphatase, and type I collagen telopeptide levels while increasing procollagen I N-terminal propeptide. Overall, the findings showed that low-dose CYC successfully prevented bone resorption while promoting bone formation, resulting in better bone quality in OVX mice.^122^ In another study, Zhang *et al.* found that walnut peptide-calcium chelate (WP-Ca) was a nutritional product made by chelating calcium ions using walnut-derived plant protein peptides as the phytonutrient. This formulation is primarily composed of walnut peptides and calcium, which provide a high calcium-binding capability while remaining thermally and digestively stable. WP-Ca considerably improves calcium absorption, with a 3.75-fold increase in intestinal uptake and calcium transport in Caco-2 cell cultures. Animal studies demonstrated that WP-Ca restored calcium insufficiency, increased trabecular number and cortical thickness, enhanced bone mechanical strength, and had a favorable relationship with healthy gut bacteria. Overall, WP-Ca is a highly bioavailable calcium supplement with promising osteoporosis prevention benefits.^123^ Similarly, Tang *et al.* made a formulation that contains calcium and vitamin D, which are micronutrients necessary for maintaining bone strength and avoiding osteoporosis. The formulation is often made as oral tablets or capsules, mixing calcium carbonate or calcium citrate with vitamin D_3_ (cholecalciferol) to increase calcium absorption. Microcrystalline cellulose, magnesium stearate, starch, and croscarmellose sodium are among the most common excipients. Post-formulation tests include hardness, friability, dissolving, and stability testing to guarantee quality and bioavailability. Clinical studies have shown that supplementing with 1200 mg calcium and 800 IU vitamin D daily dramatically lowers bone loss and fracture risk in persons over 50. Thus, calcium and vitamin D combination formulations are excellent osteoporosis prevention medications when provided correctly and consistently.^124^

### 5.2 Micronutrients

Various researchers have also done various studies utilizing micronutrients for bone tissue regeneration. For instance, Shen *et al.* developed green tea (Camellia sinensis), which contains phyto-micronutrients, such as catechins, particularly epigallocatechin gallate (EGCG) and kaempferol, with osteoprotective effects. A kaempferol-enriched green tea extract formulation (USKECSE) was created by acid hydrolysing tea leaves to increase kaempferol content while decreasing EGCG levels. This standardised extract was tested using HPLC, *in vitro* osteoblast cell assays, and *in vivo* experiments in ovariectomized rats. Post-formulation tests included the measurements of bone mineral density, bone strength, and biochemical indicators of bone production and resorption. The findings revealed that USKECSE increased bone mass and microarchitecture while remaining safe and reducing hepatotoxicity. Overall, green tea bioactive compounds promote osteogenesis, reduce osteoclast activity, and may assist postmenopausal women in avoiding osteoporosis.^144^ In another study, Li *et al.* overcame inflammation-impaired bone mending in osteoporosis, the study concentrated on creating composite microspheres as a multipurpose filler. The formulations were developed using nano-hydroxyapatite (n-HA), resveratrol (Res), and chitosan (CS). After the formulation development, several characterisations have been performed. *In vitro* studies verified sustained/controlled release of Res. Anti-inflammatory activity test (RAW264.7 cells) demonstrated a dose-dependent decrease in the expression of pro-inflammatory cytokines (TNF-α, IL-1β, and iNOS). Bone Marrow Stem Cells (BMSCs) showed improved adhesion and proliferation. Significant mineralization and the up-regulation of osteogenic markers (RUNX2, ALP, Col−1, and OCN) support osteo-differentiation. In a study of femoral condyle bone defects, an *in vitro* study using an osteoporotic rat model demonstrated improved entochondrostosis (endochondral ossification) and bone regeneration. Lastly, it has been found that the formulations are very promising for treating osteoporosis and inhibiting inflammation in the bones.^145^

Different minerals, such as zinc, copper, and boron, are used as micronutrients for bone tissue regeneration by various researchers. For instance, Wang *et al.* created a formulation including calcium, vitamin D_3_, and zinc citrate to increase calcium absorption and prevent postmenopausal osteoporosis. Vitamin D_3_ was the primary phytonutrient boosting calcium uptake, with calcium and zinc citrate serving as additional vital components of the formulation. In ovariectomized rats, the optimal dose of this Ca/vitamin D–M/Zn–M combination improved bone health by improving bone micro-architecture metrics, such as bone volume/tissue volume, trabecular number, and trabecular thickness, while decreasing trabecular separation. It also increased bone and serum calcium levels in a dose-dependent manner while effectively downregulating the osteoporosis-associated markers, M-CSFR and RANKL. Overall, this combination supplement increased calcium absorption while effectively inhibiting the development of osteoporosis, indicating therapeutic promise for postmenopausal women.^146^ In another study, Nakano *et al.* reported that NOBELZIN®, a zinc-based pharmacological formulation and an oral supplement containing 25 mg of zinc, was provided twice daily in conjunction with conventional osteoporosis therapy in older patients. This medication was administered to 122 Japanese osteoporotic patients aged 65 and older who had zinc insufficiency and were observed for a year. Serum zinc levels, bone mineral density (BMD), and bone turnover markers were examined at three time points: baseline, six months, and one year. At both time points, the medication resulted in a significant rise in BMD and serum zinc levels, as well as improved bone formation markers, and no serious adverse effects or fractures. The findings suggest that zinc supplementation might safely enhance bone health and possibly reduce fracture risk in zinc-deficient osteoporotic patients.^147^

Like zinc, Atteia *et al.* made a magnesium-boron supplementation formulation, which was utilized to restore bone health in ovariectomized rats, which were models for postmenopausal osteoporosis. Magnesium was the predominant phytonutrient due to its importance in bone mineralization, while boron operated as a supplementary mineral to assist bone structure. The study aimed to assess changes in bone mineral density and osteogenic indicators, such as calcium, phosphorus, magnesium, 25-hydroxyvitamin D, parathormone, osteocalcin, and bone alkaline phosphatase. The findings revealed that ovariectomy resulted in significant reductions in bone mineral content and changes in hormonal indicators, whereas magnesium and boron—particularly in combination—significantly improved all osteogenic measures. As a result, the formulation revealed a high potential for maintaining bone health after menopause.^148^ Similarly, Zhang *et al.* formulated an injectable phosphocreatine-grafted gelatin hydrogel (BCG) containing berberine-loaded copper-carbon dot nanozymes (BBR-CuCDs). Berberine works as a phyto-micronutrient, giving osteogenic, anti-inflammatory, and antibacterial benefits. Phosphocreatine-modified gelatin provides a supporting hydrogel matrix. Copper–carbon dots give catalytic and antibacterial capabilities through Cu^2+^ coordination. This composite hydrogel dramatically increased bone marrow stem cell proliferation, adhesion, migration, and osteogenic differentiation while simultaneously stimulating angiogenic responses in endothelial cells. It substantially inhibited osteoclast development and had potent antibacterial activity against common bone infections. In an osteoporotic rat calvarial defect model, the hydrogel enhanced bone regeneration and neovascularization, confirming its status as a safe, versatile, and promising platform for osteoporotic bone defect repair.^149^

Also, Yang *et al.* made a selenium-containing protein (Se-SP) produced from selenium-rich *Spirulina platensis* as a new formulation for osteoporosis treatment. In this formulation, selenium is the major phytonutrient, with spirulina's natural protein matrix serving as the principal carrier. Se-SP increased cellular responses compared to non-enriched spirulina by increasing calcium metabolism and alkaline phosphatase activity in MC3T3-E1 cells. In an ovariectomized mouse model, Se-SP substantially reduced bone loss by increasing bone mineral density, trabecular structure, and overall bone mass. Furthermore, Se-SP decreased inflammatory cytokines, increased osteoblast differentiation, and blocked osteoclastogenesis. These data indicate that Se-SP is a more viable and effective selenium-based therapy candidate for osteoporosis treatment.^150^ In another study, Li *et al.* formulated a selenium-based therapeutic formulation for osteoporosis, employing sodium selenite and selenomethionine as the key phytonutrients due to their significant antioxidant and immunomodulatory properties. The formulation was designed to treat retinoic acid-induced osteoporosis and contained important active constituents, such as selenium compounds. The experimental results showed that selenium administration significantly elevated serum calcium levels in both the preventive and therapeutic groups when compared to the untreated model. Serum GOT, GPT, ALP, and TRACP levels were significantly lower in the prevention group, whereas calcium and phosphorus levels increased, ALP reduced, and TRACP activity increased in the treatment group. Overall, selenium-based formulations may help prevent osteoporosis and contributes to its management by enhancing bone metabolism and mineralization.^151^

The preclinical *in vivo* performance of the novel delivery system for osteoporosis management may be significantly more effective. However, there are substantial translational barriers that must be overcome in order to transition these formulations from the laboratory to the market. Three primary obstacles are identified by a critical assessment of the current landscape: economic viability, formulation stability, and scalability. Effective control over thermodynamic and mechanical parameters is necessary for the industrial scale-up of complex, multi-phase systems. Different processing variables are present in specialized manufacturing techniques, which are highly sensitive. The stability and efficacy of the formulation are influenced by these variables. These variables can be eliminated through the use of multi-component process optimization techniques. The long-term physical and chemical stability of these formulations is an additional constraint. These innovative carriers contain phytonutrients that have the potential to undergo photodegradation and oxidation. The formulation matrix can be further complicated, but these limitations can be surmounted through the use of specialized excipients or advanced lyophilization techniques. Finally, the economic and regulatory concerns must be resolved. The cost of formulations may be elevated in comparison to that of standard vitamin or mineral tablets due to the excipient costs, specialized manufacturing apparatus, and complex quality control assays. Production costs may continue to be a substantial impediment to the widespread clinical adoption of chronic disease management systems, such as osteoporosis, unless manufacturing workflows are optimized and standardized.

## 6. Clinical evidence and other therapeutic implications

Despite significant preclinical studies indicating the beneficial impact of plant-derived macronutrients and micronutrients on bone health *via* the modulation of osteoblast differentiation, osteoclast activity, oxidative stress, inflammation, and bone remodeling pathways, the application of these findings in clinical practice continues to be a significant area of research. Recent clinical studies have assessed the effectiveness of plant-derived nutrients and bioactive compounds, such as calcium-rich plant foods, soy isoflavones, plant proteins, vitamin K, magnesium, polyphenols, and phytoestrogens, in enhancing bone mineral density, diminishing bone turnover, and decreasing fracture risk in osteoporotic and postmenopausal populations (Table 3).

**Table 3:** Clinical evidence of phytonutrients used in osteoporosis

Plant scientific name	Part/type of preparation	Macro or micro nutrients present in the plant parts	Country	Study design	Participant details	Outcome	Ref.
*Actaea racemosa* L.	Rhizome/capsule	This part contains calcium and magnesium	Germany	Double-blinded randomized controlled trials	62 postmenopausal women between the ages of 40 and 60	Elevated BALP with no impact on liver enzymes	157
*Actaea racemosa* L.	Rhizome/extract	Rhizome contains calcium and magnesium	Spain	Randomized controlled trials	72 postmenopausal women, with a mean age of 55.4 ± 5.5 years	Low telopeptides, high BALP, and no impact on osteoblasts	158
*Allium cepa* Linn.	Bulbous/juice	This part contains magnesium and calcium	Republic of China, Taiwan	Randomized controlled trials	30 healthy participants (12 males and 18 females) between the ages of 40 and 80	Low free radicals, low ALP, high TEAC, and high BM	159
*Asparagus racemosus* Willd.	Root/capsule	Roots mainly contain calcium	UK	Parallel, double-blinded randomized controlled trials	20 male participants and no female participants, with a mean age of 70.1 ± 2.1 years (range: 66.8 ± 1.5 years)	Did not alter the plasma markers: P1NP and β-CTx enhance muscular contraction and function	160
*Camellia sinensis* L*.*	Leaves/capsule	Leaves contain protein, carbohydrate, calcium and magnesium	USA	Double-blinded randomized controlled trials	140 overweight/obese women between the ages of 50 and 70 (F/M: 140/0)	No impact on BMD	161
*Camellia sinensis* L.	Leaves/capsule	Leaves contain protein, carbohydrate, calcium and magnesium	USA	Randomized controlled trials	171 postmenopausal osteoporotic women between the ages of 50 and 70 (F/M: 171/0)	High BAP and BAP/TRAP ratio in the exercise groups for GT and TC	162
*Cissus quadrangularis* L.	Leaves/capsule	Leaves contain calcium, magnesium, carbohydrate, and protein	Thailand	Randomized controlled trials	134 women over 40 (F/M: 134/0)	Low % P1NP in treatment groups and no impact on BMD	163
*Cornus mas* L.	Fruit/capsule	Fruit contains carbohydrate, protein, calcium, magnesium, zinc, and copper	Iran	Double-blinded randomized controlled trials	The mean age of the 84 women in the research ranged from 52.57 ± 0.64 to 53.43 ± 0.49 years (F/M: 84/0)	Low hs CRPC, PTH, and BALP. No impact on TC and OC	164
*Glycine* max (L.) Merr.	Bean/milk	This part contains protein, carbohydrate, fat, calcium, magnesium, zinc, and copper	Denmark	Double-blinded randomized controlled trials	107 postmenopausal women (F/M: 107/0) between the ages of 40–75	BMD and BMC of the low lumbar spine in the placebo and combined treatment groups	165

While several plant-derived macro- and micronutrients have shown positive benefits to bone health, there is a limitation of direct comparison studies assessing their relative usefulness. Future comparative clinical studies are necessary to identify the most effective dietary strategies for the prevention and treatment of osteoporosis.^59^

These plant-derived macro- and micronutrients have a recognized function in the prevention and treatment of osteoporosis. While these plant-derived macro- and micronutrients may provide extensive health advantages, they operate *via* common molecular pathways associated with inflammation, oxidative stress, tissue remodeling, and cellular metabolism. Calcium enhances cardiovascular health by activating calcium-sensing receptors (CaSRs) in vascular smooth muscle cells, thereby regulating vascular tone and blood pressure, while also facilitating skeletal muscle performance.^152^ Magnesium boosts insulin signaling, improves mitochondrial bioenergetics, and inhibits NF-κB-mediated inflammation, indicating possible advantages in type-2 diabetes, heart disease, and metabolic syndrome.^153^ Plant-derived proteins similarly stimulate the IGF-1 and mTOR signaling pathways, which are essential for muscle protein synthesis and tissue regeneration, underscoring their potential significance in sarcopenia, frailty, and cachexia.^154^ The activation of osteocalcin and matrix Gla protein by vitamin K could help in the prevention of vascular calcification and inflammatory conditions, while vitamin C facilitates collagen production, antioxidant protection, wound healing, and immunological response *via* Nrf2-mediated mechanisms.^155^ Moreover, folate modulates homocysteine metabolism and DNA methylation, processes associated with cardiovascular and neurological disorders. Collectively, they indicate that the molecular pathways by which plant-based nutrients enhance bone health may potentially aid in the prevention and treatment of several chronic age-related illnesses, necessitating more clinical explorations.^156^

## 7. Conclusion and future directions

Osteoporosis is a considerable global health concern, especially among post-menopausal women and the elderly. It is characterized by an imbalance between bone creation and resorption, leading to a gradual loss of bone mass and an elevated risk of fractures. This study emphasizes the crucial function of plant-derived macro- and micronutrients in preserving bone health and reducing the development of osteoporosis. Accumulated data from several study methodologies demonstrate the significant impact of phyto macro and micro nutrients, including proteins, peptides, carbohydrates, calcium, magnesium, zinc, and selenium. These critical nutrient components affect bone health and underscore their capacity to maintain bone integrity while regulating the dynamic processes of bone remodeling and homeostasis *via* many interrelated molecular pathways. These nutrients modulate essential signaling networks implicated in bone remodeling, such as RANKL/RANK/OPG, NF-κB, Wnt/β-catenin, PI3K/Akt, and ERK/MAPK pathways, thereby affecting osteoblast differentiation, osteoclast activity, mineralization, and inflammatory responses. Recent data from various research approaches, including *in vivo* and *in vitro* models, as well as clinical trials, demonstrate that phyto supplements containing these nutrients considerably increase bone health, especially in post-menopausal osteoporotic circumstances. These findings demonstrate that plant-based macro and micronutrients provide a complete, safe, and sustainable approach for both the preventive and potential adjunctive therapy of osteoporosis.

Furthermore, the use of innovative delivery systems has significantly enhanced the bioavailability and targeted administration of these organic supplements. There are still a number of knowledge gaps despite significant advancements. Future research must emphasize methodically constructed, long-term randomized clinical trials to determine appropriate doses, treatment durations, and population-specific guidelines for plant-based nutritional therapies. This will also enhance their therapeutic effectiveness, indicating increased potential for future medicinal uses. Further research is also necessary in developing fields, including plant-derived bioactive peptides, nutrient-receptor structure–activity connections, and sophisticated formulation techniques. Addressing these gaps will enable phytonutrient-based medicines to evolve into evidence-based, integrated solutions for the worldwide management of osteoporosis.

## Author contributions

Dr G. D., Mr M. J. and Dr A. S. conceptualized and structured the study. Ms M. R., Dr G. D., and Mrs A. D. conducted the methodology and research. Ms M. R. and Ms S. S. R. prepared all the schematic diagrams, and Dr G. D., Dr A. S. and Dr B. D. reviewed and critically revised the manuscript. All authors contributed equally to its drafting and have read and consented to its submission.

## Conflicts of interest

The authors declare no conflicts of interest.

## Data Availability

No primary research results, software or code have been included, and no new data were generated or analysed as part of this review.
